# Fiber-Reinforced Composites for Dental Applications

**DOI:** 10.1155/2018/4734986

**Published:** 2018-11-01

**Authors:** Andrea Scribante, Pekka K. Vallittu, Mutlu Özcan

**Affiliations:** ^1^Unit of Orthodontics and Pediatric Dentistry, Section of Dentistry, Department of Clinical, Surgical, Diagnostic and Pediatric Sciences, University of Pavia, Italy; ^2^Department of Biomaterial Science and Turku Clinical Biomaterials Centre (TCBC), Institute of Dentistry, University of Turku, Turku, Finland; ^3^City of Turku, Welfare Division, Turku, Finland; ^4^University of Zurich, Center for Dental and Oral Medicine, Dental Materials Unit, Clinic for Fixed and Removable Prosthodontics and Dental Materials Science, Zurich, Switzerland

Fiber-reinforced composites (FRCs) are composite materials with three different components: the matrix (continuous phase), the fibers (dispersed phase), and the zone in between (interphase). FRC materials present high stiffness and strength per weight when compared with other structural materials along with adequate toughness. FRCs have been used for numerous applications in various engineering and biomedical fields for a long time. The reinforcement of dental resins with either short or long fibers on the other hand has been described in literature for more than 40 years [1]. FRCs based on carbon, polyaramid, polyethylene, and glass have been largely studied and among all, glass fibers of various compositions are more commonly applied as restorative and prosthetic materials [2, 3].

FRCs have been intensively investigated with a particular emphasis on mechanical properties such as fracture toughness, compressive strength, load-bearing capacity [4], flexural strength [5], fatigue resistance [6], fracture strength [7] or on the effect of layer thickness [8], bacterial adhesion [9], adhesion of fibers for various dental applications, such as long fibers [10], nets [11], and posts [12]. From clinical perspective, FRCs have been investigated for different clinical applications in prosthodontics, such as replacement of missing teeth by resin-bonded adhesive fixed dental prostheses of various kinds [13], reinforcement elements of dentures or pontics [14], and direct construction of posts and cores [15]. In other disciplines of dentistry, such as orthodontics FRCs have been suggested as active and passive orthodontic applications (i.e., anchorage or en-masse movement units) and postorthodontic tooth retention [16] and in periodontology for splinting mobile teeth in an attempt to prolong tooth extraction [17].

With the introduction of new technologies, nanofillers, resin matrices, fibers, adhesion protocols, and application techniques, the design principles of FRC devices need further understanding which open new fields of research both preclinically and clinically [18]. On the basis of these considerations, BioMed Research International prepared the present special issue in an attempt to explore these new variables related to FRCs.

Guest editors do hope that this special issue would be interesting for the readers of the journal and wish that the present work could help both clinicians and researchers to understand FRC applications and properties.
